# Low concentration of serum immunoglobulin G is associated with pre-weaning diarrhea in young mink kits (*Neovison vison*)

**DOI:** 10.1186/s13028-019-0461-5

**Published:** 2019-06-10

**Authors:** Ronja Mathiesen, Mariann Chriél, Tina Struve, Peter Mikael Helweg Heegaard

**Affiliations:** 1 Innate Immunology Group, Section for Protein Science and Biotherapeutics, Department of Biotechnology and Biomedicine, Technical University of Denmark, Kemitorvet Building 204, 2800 Kgs. Lyngby, Denmark; 2 Division of Diagnostics & Scientific Advice-Diagnostic & Development, National Veterinary Institute, Technical University of Denmark, Kemitorvet Building 204, 2800 Kgs. Lyngby, Denmark; 3 Kopenhagen Fur, Langagervej 60, 2600 Glostrup, Denmark

**Keywords:** Immunoglobulin G, Mink kits, *Neovison vison*, Pre-weaning diarrhea

## Abstract

**Background:**

Pre-weaning diarrhea (PWD) is a severe syndrome, with world-wide occurrence, affecting farmed mink (*Neovison vison*) kits during the lactation period. Kits affected by PWD often display clinical signs such as: yellow-white diarrhea, greasy skin, and dehydration. In severe cases the kits eventually die. It is common practice to treat PWD using antimicrobials; however the effect is not well documented. Due to the multifactorial etiology of PWD vaccine development is not feasible. The role played by the immune status of the mink kits with respect to their susceptibility to PWD is not well studied. To elucidate the possible association between PWD and total IgG serum concentration in young kits we analyzed blood collected from kits from 100 litters on two mink farms during the same breeding period, one farm being a case farm with high prevalence of PWD, and the other being a control farm with no cases of PWD.

**Results:**

Kits affected by PWD had a significantly reduced weight gain compared to unaffected control kits. Litters born later in the breeding period came down with PWD at an earlier age than litters born at the start of the breeding period. We found that PWD affected kits had significantly lower concentrations of serum IgG compared to unaffected kits at 13–15 days of age (the last blood sampling point of the study).

**Conclusion:**

The results in this study suggest that PWD affected kits less efficiently absorbed IgG from maternal milk or had a lower intake of maternal milk, potentially contributing to the exacerbation of disease. A lower intake of IgG and/or less absorption from maternal milk could also pre-dispose kits for PWD. Future studies will be needed to elucidate if the circulating level of IgG is directly related to protection against disease and to investigate if administration of IgG could be helpful in alleviating and/or preventing PWD in mink kits.

## Background

A year on a mink farm in the Northern Hemisphere begins after pelting at the end of November and start December. Only mink selected for breeding continue on to the next year [1]. Due to the photoperiod and climate of the Northern Hemisphere all mink come into heat once a year in March [2]. Mink kits are born in late April to mid-May and after 4 weeks of lactation they start eating by themselves. During the lactation period antimicrobials are frequently used on the dams and/or kits, increasing the risk of antimicrobial resistance in bacteria [3, 4]. This increase in use of antimicrobials during the lactation period could be due to an increased incidence or increased awareness, and/or severity of pre-weaning diarrhea (PWD) [5, 6]. PWD is common on mink farms and the onset is usually around 1–4 weeks after parturition; however both morbidity and mortality varies between farms and breeding periods [7–9]. Finding clinical signs in more than 15% of the litters on a farm is considered a severe outbreak [10]. Affected kits present signs such as; a profuse yellow/white foamy diarrhea, dehydration, greasy skin as a result of increased secretion from the cervical apocrine glands in the neck region, a red and swollen perianal region, and distressed vocalization [8, 11]. In severe cases, PWD can lead to dehydration and eventually death. Furthermore, kits affected by PWD have a lower body weight compared to age-matched healthy control kits [12]. Because of the resource demanding consequences and economic losses associated with PWD a lot of interest has been directed towards understanding the syndrome and finding the specific cause in order to prevent or cure PWD. However, the syndrome is considered to have a multifactorial etiology, with no defined cause, and although some enteropathogens, including both bacteria and virus have been implicated, it has proven to be difficult to pinpoint specific pathogens of major significance for developing PWD [9, 13]. Some risk factors associated with PWD include; the birth date of the kits-with a higher risk associated with being born late in the breeding period [7], as well as the age of the dam with first year dams having a higher incidence of affected kits than second year dams [5, 7]. It has been shown by litter mixing experiments that the dam is an important factor in contracting PWD [8]. The fact that older dams have a lower risk of getting kits affected by PWD could indicate that the maturity of the maternal immune system could be important and that bolstering the mink kits’ own immune system could be part of the solution. The predominant immunoglobulin found in mink milk is immunoglobulin G (IgG) [14]. Mink kits are born with an immature immune system and with a very low serum concentration of IgG [14, 15]. It is vital for the kits that they absorb the IgG from the dams’ colostrum and milk after birth, which provides an defense system against a wide range of microbes and convey passive immunity until the mink kits start producing IgG by themselves 7–8 weeks after parturition [14]. Mink kits are able to transfer IgG from the dams’ milk to the circulation until they are at least 47 days old [14, 16], which is in contrast to other farm-raised animals, like ruminants and pigs, where the gut-passage of IgG closes 24 h after parturition [17]. The principle of passive immunization with antibodies delivered from mother to offspring has been demonstrated more than a 100 years ago [18]. The protective effect of giving immunoglobulins towards bacteria to pigs [19, 20], and against virus to ferrets [21, 22] and mink kits [23] has been reported previously. The protective role of passive immunization by maternally transferred IgG with regards to PWD in mink kits has not been investigated previously. The objective of this study was to determine if there was an association between mink kit serum IgG concentration and the development of PWD.

## Methods

### Animals

A longitudinal study of a total of 100 first-year American mink (*Neovison vison*) breeding dams and their offspring was performed during the pre-weaning period (April–May 2017) at two commercial certified mink farms located in Zealand, Denmark: one case farm (56% of the 50 litters were affected by PWD at day 13–15) and one control farm (no PWD in the 50 litters). The farms were certified free from Aleutian mink disease virus (AMDV) [24]. The control farm vaccinated all mink in the summer period (June) with a commercial mink vaccine against mink distemper and mink enteritis virus (ATC-code#QI20CH, Biovet Aps, Fredensborg, Denmark), while the case farm did not vaccinate. The farms were voluntarily enrolled and the mink dams were selected based on age (1-year old dams), litter size (n = 6–9), and both dark and light colored litters were included in the study. One-year old dams were selected based on the increased risk of PWD [5, 7]. The mink kits on the case farm showed typical clinical signs of PWD, a profuse yellow/white foamy diarrhea, dehydration, greasy skin as a result of increased secretion from the cervical apocrine glands in the neck region, and a red and swollen perianal region [8, 11, 25], while no litters on the control farm showed any of these signs. All the mink in this study were housed in separate cages with conventional nest boxes and the adult mink dams were fed a commercial mink diet (Sjællands Pelsdyrfoder, Højby Sj., Denmark) with free access to water. All the litters were weighed when included in the study and every second day thereafter until the termination of the study. The total weight of the whole litter was rounded to the nearest tenth of a gram and the mean mink kit body weight was obtained by dividing total weight of the litter by the total number of kits in the litter. The mink kits were scored (present/absent) for clinical signs of PWD syndrome in regards to presence of red and swollen perianal region, signs of dehydration, “greasy” neck region, and if there was defecation; the consistency (runny) and color (beige-white) [25]. All signs had to be present for the kits to be considered affected by PWD.

### Sample collection

Four groups with varying numbers of litters were formed according to birth dates (1–4, see Table 1).

**Table 1:** Group scheme depicting the number assigned to each group of litters born on different dates

Farm	Group	No. litters	Date of birth (year 2017)
Control	1	10	April 26th
Case	1	10	April 27th
Control	2	10	April 28th
Case	2	16	April 29th
Control	3	16	April 30th
Case	3	14	May 1st
Control	4	14	May 2nd
Case	4	10	May 3rd

Table 2 summarizes the sampling scheme used for each farm. Sampling started when the mink kits in each group were 1 day old with the blood sampling of all adult dams and one mink kit from each of the 50 litters. Repeated blood and milk samples were taken from 10 selected dams (group 4) when the kits were 3, 5, and 7 days old as well as blood samples from four kits (two kits from two litters). Finally, when the kits were 13–15 days old, milk was sampled from the same 10 dams and blood samples were collected from all 50 dams and two kits from each litter. Dams and their remaining kits were returned to the farmers after the last sampling day.

**Table 2:** Blood and milk samples collected at different ages of the mink kits

Farm	Kit age (days)	No. maternal blood samples	No. milk samples	No. kit blood samples
Control	1	50	–	50
Case	1	50	–	50
Control	3/5/7	10	10	4^a^
Case	3/5/7	10	10	4^a^
Control	15	50	10	100
Case	13–15	10/40^b^	10	99^c^

^a^Blood from two mink kits from two litters were collected

^b^Sampling of group 4 (n = 10 litters) and their offspring ended on day 13, while the rest of the litters (n = 40) were sampled until day 15 (on the case farm)

^c^One litter had blood collected from only one kit

Dams were restricted in cages and blood sampled via vena cephalica. Milk was obtained by first injecting the dams with 0.5 mL of oxytocin (10 IE/mL, #444687, MSD Animal Health, Copenhagen, Denmark) to stimulate milk flow [26] and then milking by hand. Milk from different glands was combined. Blood samples were obtained from mink kits euthanized with CO or CO_2_ and then bled except for the last sampling time point (13–15 days old) where most kits were blood sampled via vena jugularis and then returned to the litter. Blood was allowed to clot and serum was obtained after centrifugation at 4000*g* for 15 min at 4 °C. Both milk and serum samples were stored at − 20 °C until analysis.

### IgG quantification in serum and milk samples

A validated quantitative sandwich ELISA was used for quantification of mink IgG in serum and milk samples [15]. The capture antibody was a commercially available (Sigma-Aldrich, St. Louis, MO, USA), mink IgG cross-reactive polyclonal goat anti-ferret IgG antibody and detection was accomplished by the same antibody conjugated with horseradish peroxidase (also commercially available). Samples were run in double determinations and specific buffer conditions were applied for serum and milk samples, respectively [15]. Calibration was performed using an in-house purified mink IgG standard. The detection limit was 5 ng/mL for serum samples and 1 ng/mL for milk samples.

### Statistics

Data were analyzed and graphed in GraphPad Prism version 7 (GraphPad Software, San Diego, California, USA, http://www.graphpad.com). Normality was analyzed for all data using the Shapiro–Wilk test of normality, indicating non-normal distribution of data. The Mann–Whitney U non-parametric test of significance was used to test for differences in median kit body weight gain and kit serum IgG concentrations from the control farm and the case farm at different time-points. The difference between maternal serum IgG concentrations at different time-points was tested for significance with the Kruskal–Wallis test and Dunn's multiple comparison post hoc test. The correlation between maternal serum and milk IgG concentrations was analyzed by the Spearman rank correlation test. Results are presented as median ± interquartile range (IQR). Differences were considered significantly different at P < 0.05. Outliers (n = 2) were identified using the ROUT method (Q = 1%) and removed [27].

## Results

**Fig. 1:**
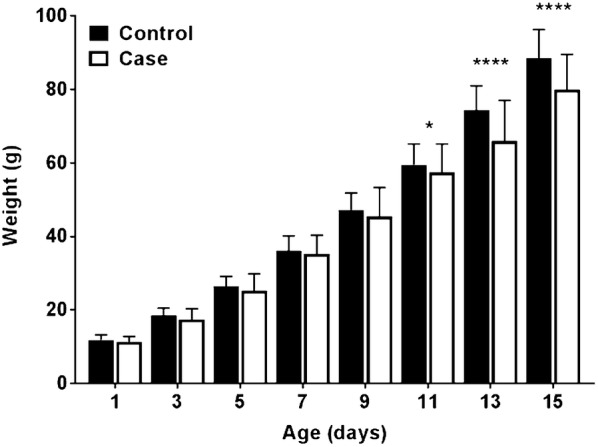
The median kit body weight of control (black columns) vs. case (white columns) mink kits. All groups (1–4) on the control farm and groups 1–3 on the case farm were weighed until day 15, while group 4 on the case farm was weighed until day 13. Statistical significance of differences between the two farms was determined by the Mann–Whitney U test (for nonparametric variables) (*P < 0.05; ****P < 0.0001). Bars indicate the median ± IQR

The median kit body weight for both farms when the kits were 1 day old was 11.9 g (25th and 75th percentiles 11.0–13.2 g) on the control farm and 11.3 g (25th and 75th percentiles 9.5–12.8 g) on the case farm (n = 401 for both farms, Table 3). When the kits were 1–9 days old there was no significant difference observed in the median kit body weight between the case and the control farm (Fig. 1 and Table 3). However, the median body weight of the kits from the case farm was consistently different than that of the control farm from day 3 and onwards and this difference increased with the age of the kits (Fig. 1 and Table 3). The observed median kit body weight (Fig. 1) was significantly different between control (median 59.5 g, 25th and 75th percentiles 53.4–65.2 g) and case kits (median 57.4, 25th and 75th percentiles 46.0–65.2 g) when the kits were 11 days old (P < 0.05) and until the end of the sampling on day 15 (median on the control farm 88.5 g vs. 79.9 g on the case farm; P < 0.0001).

**Table 3:** Mink kit body weight results from the two farms

Kit age (days)	Control farm				Case farm			
	No. weighed kits	Median kit body weight (g)	25th percentile (g)	75th percentile (g)	No. weighed kits	Median kit body weight (g)	25th percentile (g)	75th percentile (g)
1	401	11.9	11.0	13.2	401	11.3	9.5	12.8
3	334	18.5	16.3	20.5	343	17.4	15.6	20.3
5	327	26.4	23.4	29.1	331	25.2	22.7	29.8
7	314	36.1	31.1	40.2	323	35.2	30.3	40.3
9	306	47.2	41.6	51.8	321	45.4	37.0	53.3
11	306	59.5	53.4	65.2	316	57.4	46.0	65.2
13	306	74.4	67.5	81.0	307^a^	65.9	53.7	77.0
15^b^	305	88.5	80.1	96.3	256	79.9	66.0	89.6

^a^Kits in case litters (PWD affected) decreased after day 13

^b^All groups (1–4) on the control farm and groups 1–3 on the case farm were weighed until day 15, while group 4 on the case farm was weighed until day 13

**Fig. 2:**
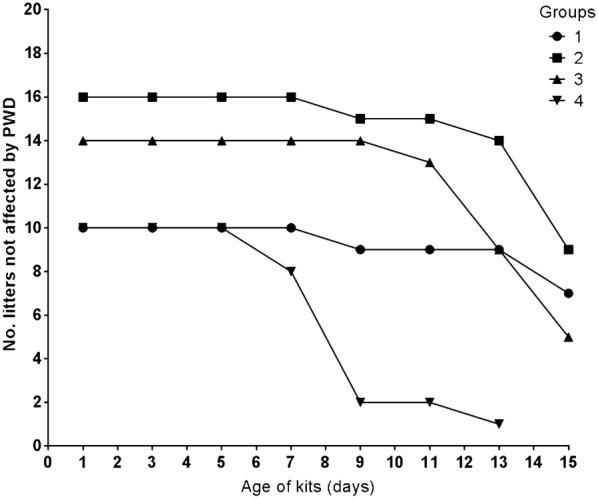
Incidence of pre-weaning diarrhea (PWD) in the four different sampling groups on the case farm. Each sampling group (1–4) had different number of litters (group 1 = 10, group 2 = 16, group 3 = 14, and group 4 = 10) and birth dates of the kits as seen in Table 1. The scoring of PWD was done every other day. However, due to the different birth dates of the mink kits the scoring of PWD ended at different ages of the kits (day 15 for group 1–3 and day 13 for group 4)

The later in the breeding period the mink kits were born (group 4) the earlier onset of PWD was observed as shown in Fig. 2 where two out of ten litters in group 4 were already affected by PWD when the kits were 7 days old. This number of affected kits in group 4 increased until day 9. The other groups (group 1–3) on the case farm were affected by PWD from day 9–11 and as shown in Fig. 1 and Table 3 there was a consistent decrease in body weight gain during the same time period.

**Fig. 3:**
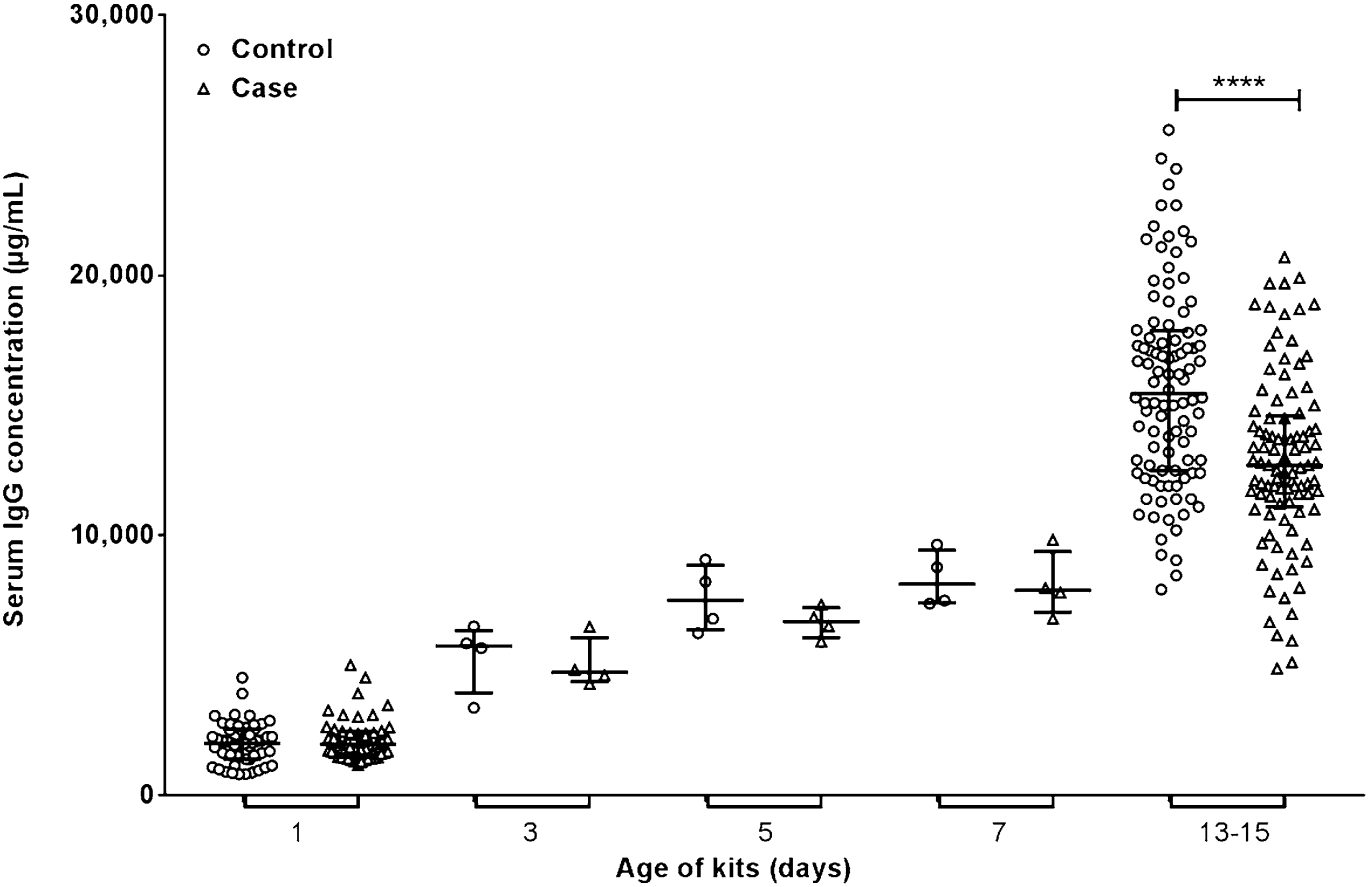
Mink kit serum immunoglobulin G (IgG) concentrations from 1 to 13–15 days of age on the control farm (circles) vs. the case farm (triangles). n = 50 (day 1), n = 4 (days 3, 5, and 7) and n = 100 (day 15, control farm) and 97 (day 13–15, case farm, two outliers were removed (ROUT method) [27] and one litter was missing kits). Significance of difference between serum IgG concentrations was analyzed by the Mann–Whitney U test **(**for nonparametric variables). ****P < 0.0001. Bars indicate the median ± IQR

Serum IgG concentrations for mink kits at day 1, 3, 5, 7, and 13–15 postpartum were determined by sandwich ELISA (Fig. 3). There was no significant difference in kit serum IgG concentration between the control farm (circles) and the PWD case farm (triangles) at days 1–7 (Fig. 3). Although Fig. 3 showed no difference in serum IgG concentration between farms when the kits were 1 day old, the figure does suggest that the serum IgG concentration was lower in mink kits affected by PWD compared to control kits when the kits were 3, 5, and 7 days old. It should be noted that during these time points there were only two kits from two different litters included, amounting to four kits from each farm. At 13–15 days, the difference in mink kit serum IgG concentration was statistically significant (P < 0.0001), with mink kits affected by PWD having a lower serum IgG concentration than kits from the control farm. The median serum IgG concentration of the control mink kits was 15,450 µg/mL ± 5375 µg/mL when the kits were 15 days old, while the median serum IgG concentration for the case mink kits at day 13–15 was 12,700 µg/mL ± 3500 µg/mL.

There was no significant difference observed in the serum IgG concentration between dams from the case and the control farms during any given time point (data not shown). However, when pooling all the dams’ serum IgG concentration results from the two farms there was a significant difference (P < 0.05) between day 1 and day 13–15 and also day 3 and day 13–15 (data not shown). Milk IgG concentration remained constant throughout the sampling period with no significant differences between farms and days (data not shown). In addition, Spearman rank correlations for nonparametric data showed no correlation between maternal milk and serum IgG concentrations (P = 0.08, not shown).

## Discussion

Based on a previous observation on mixed litters in which kits moved from their original litter, which later got affected by PWD, developed PWD in the “new” litter, while the new littermates did not [8], we hypothesized that maternal factors may be important for the susceptibility of kits to PWD. One such potentially important factor could be maternal IgG and its efficiency of transfer from dam to offspring. In contrast to other farm-raised animals, like the ruminants and pigs, in which intestinal transport of IgG takes place only within the first 24 h after parturition [17], the mink kit intestine allows uptake of maternal IgG until 4–5 weeks after parturition [14]. The kits’ own IgG production does not start until 7–8 weeks after parturition [14] and we therefore chose to study the maternally derived IgG in mink kit serum from birth until the kits were 13–15 days old. We have previously established that the serum IgG concentration in healthy mink kits reaches a constant level not differing much between kits at day 8 after birth and onwards [15]. It is therefore assumed that there should be no significant difference between serum IgG concentrations at day 13 versus day 15 postpartum in healthy mink kits.

As shown in Fig. 1 mink kits suffering from PWD had a significantly lower weight from day 11 compared to the control farm confirming previous results [12]. A litter size above seven kits decreases the growth rate [28], however as indicated in Table 3 the number of kits did not differ between the control and the case farm in this study up to and including day 15—as group 4 on the case farm was not included in the last weighing session there was a lower total number of kits on day 15. Also, the incidence of PWD was increasing on the case farm around day 11 for groups 1–3 (Fig. 2) suggesting that PWD is indeed the cause of the lower weight of the kits on the case farm.

Furthermore, we found the incidence of PWD to be dependent on the date of birth; the later in the breeding period mink kits were born, the younger they were when they got affected by PWD, i.e. litters from group 4 had a faster onset of PWD compared to the other litters on the case farm (Fig. 2). It should be considered that group 4 was also the most intensely handled group as it was the only group in which both maternal blood and milk samples were taken at day 3, 5, 7, and 13. This could theoretically increase the susceptibility of the kits to disease due to maternal stress. Furthermore, a build-up of pathogens from the other litters could increase the risk of PWD in the late born litters due to their low concentration of serum IgG. Recommendations for on-farm management of PWD should include better hygiene regarding handling of sick and healthy kits at different times. However, a recent study done by Birch et al. [5] showed that there was no difference in hygiene precautions and management between the case and control farms, indicating that these factors are not associated with development of PWD. There was no difference in serum IgG concentration between the mink kits from the control and case farm on day 1, 3, 5, and 7. There was, however, a tendency at these time points towards a lower IgG serum concentration in case kits compared to control kits. At day 1 the sample size was 50 mink kits per farm, however at day 3, 5, and 7 the sample size was low with serum IgG concentrations measured in only two kits from two litters (n = 4) out of 50 litters. Analyzing more kits would strengthen the results regarding the concentration of IgG in kit serum during these time points and increase the knowledge on the possible impact of low IgG serum concentration on the risk of developing PWD later in the pre-weaning period. When the kits were 13–15 days old, the IgG serum concentrations in case kits were significantly lower than in control kits (P < 0.0001, Fig. 3). To investigate if this difference in mink kit serum IgG concentrations could be due to different IgG concentrations in the dams we analyzed the IgG concentration in both blood and milk of the dams. As mammary secretions of IgG to the milk is largely dependent on IgG from the circulation [29] we firstly analyzed blood samples taken from the dams on day 1, 3, 5, 7, and 13–15, and found no significant difference between the farms. There was no difference in milk IgG concentrations either (data not shown). There may still be differences in the spectrum of antigenic specificities covered by the maternal IgG pools involved, i.e. maternal IgG on the control farm could have on average a broader specificity against pathogens compared to the maternal IgG pools of the case farm. Older dams have possibly developed a circulating IgG pool with a broader pathogen coverage, which could explain why there is an increased risk of PWD among first year dams compared to second year dams [5, 7].

In summary, mink kits affected by PWD had reduced serum IgG concentrations after 13–15 days of age, which was not associated with a lowered IgG concentration in maternal milk as there was no significant difference in milk IgG concentrations between control and case farm. The lower levels of circulating IgG could be due to a lower consumption of milk and/or impaired efficiency in taking up the ingested maternal milk IgG and could contribute to increased susceptibility to PWD. It is not clear if the circulating level of IgG is directly important for protection against disease or if it is simply an indicator of the intestinal level of maternal IgG providing local immune protection against PWD. Future studies should investigate the causality i.e. does a low serum IgG concentration lead to a higher susceptibility to disease or is the decreased IgG concentration simply an effect of a lowered intake of maternal milk/lowered efficiency of IgG uptake by PWD affected animals. Orally administration of IgG has been shown in piglets to reduce the bacterial load in experimental intestinal infection [19]. As PWD is a multifactorial disease, administering a pool of IgG with a broad specificity might provide protection by counteracting a range of pathogens important for PWD, without having to identify the specific cause of the syndrome. Our results show an association between PWD and low serum concentrations of total IgG. It remains to be tested if giving broad-specificity IgG as a feed supplement to mink kits could possibly increase survival rate and welfare by decreasing the incidence and severity of PWD.

## Conclusions

PWD is still a large issue on many farms in fur-producing countries and as it is associated with a multifactorial etiology finding a possible cure/treatment is challenging. Our results show that the serum IgG concentration is lower in mink kits affected by PWD compared to the control kits, when they are 13–15 days old. This suggests that PWD-affected kits less efficiently absorbed IgG from maternal milk or had a lower intake of maternal milk as their weight was also lower compared to the control kits. This reduced uptake of milk IgG could potentially contribute to an exacerbation of the disease. Future studies will be needed to elucidate if the circulating level of IgG is directly related to protection against disease and to investigate if immunoglobulin supplementation could be helpful in alleviating and/or preventing PWD in mink kits.

## Data Availability

The datasets used and/or analyzed during the current study are available from the corresponding author on reasonable request.

## Abbreviations

ELISA: enzyme-linked immunosorbent assay
IgG: immunoglobulin G
IQR: interquartile range
mL: milliliter
PWD: pre-weaning diarrhea
